# Sexual Harassment and Gender-Based Harassment Among Teaching and Research Staff at a Public University in Northwestern Spain: Prevalence and Predictors

**DOI:** 10.3390/bs16030466

**Published:** 2026-03-21

**Authors:** Yolanda Rodríguez-Castro, Mar Fernández-Cendón, Rosana Martínez-Román, Xosé María Mahou-Lago

**Affiliations:** 1 Faculty of Education and Social Work, University of Vigo, 32004 Ourense, Spain; rosana.mr@uvigo.gal; 2 PhD Program in Creativity and Sustainable Social Innovation, University of Vigo, 36005 Pontevedra, Spain; marcendon@uvigo.gal; 3 Faculty of Public Management and Administration, University of Vigo, 36005 Pontevedra, Spain; xmahou@uvigo.gal

**Keywords:** sexual harassment, sex-based harassment, technology-facilitated sexual harassment, teaching and research staff

## Abstract

The aim of this study is twofold: (a) to identify the prevalence of sexual harassment and sex-based harassment among teaching and research staff (TRS) at a public university; and (b) to examine the predictive capacity of sociodemographic variables and prior harassment experiences on the frequency of different forms of sexual victimization (SEQ): gender harassment, unwanted sexual attention, and sexual coercion. A total of 425 TRS members participated (48.9% women, 50.6% men, 0.5% not identified; mean age = 45.88, *SD* = 22.2), all affiliated with a public university in northwestern Spain. Findings showed that female TRS explicitly self-identified as victims of sexual harassment and gender-based harassment within the university. Overall mean scores on the three SEQ subscales were low, yet women reported significantly higher levels of gender harassment and unwanted sexual attention. Female TRS also showed higher levels of technology-facilitated sexual harassment compared with their male counterparts. Hierarchical regression analyses indicated that prior sexual victimization and technology-facilitated harassment were the strongest predictors across all SEQ dimensions. Unwanted sexual attention and TFSV predicted sexual coercion, whereas higher professional rank was associated with a reduced risk within this university. In conclusion, this public university requires well-disseminated and trusted protocols that explicitly address digital forms of sexual violence, alongside sustained preventive programs aimed at reducing revictimization.

## 1. Introduction

The focus on studying sexual harassment and sex-based harassment in the workplace emerged in the 1970s, particularly in the United States, which contributed to its visibility and triggered a proliferation of international research aimed at quantifying the phenomenon (Willness et al., 2007). Most international studies underscore the high incidence of sexual and sex-based harassment in the workplace (United States Equal Employment Opportunity Commission (EEOC), 2024). In fact, recent studies indicate that one in four women has experienced some form of sexual violence, such as sexual harassment, during their professional career (Debnath et al., 2025; Organisation for Economic Co-operation and Development, 2025).

### 1.1. Conceptual Definition and Legislation

There is considerable variability in the definitions of sexual harassment and sex-based harassment, primarily due to the diverse approaches adopted in legislative, psychological, and other fields (Herrera et al., 2016). In Europe, the turning point was marked by the Council of Europe (2011) in Istanbul on preventing and combating violence against women. Spain signed the convention and ratified it in 2014. The Spanish Constitution (1978) enshrines the principle of equality between men and women; however, sexual harassment was not recognized as a criminal offense until the enactment of Organic Law 10/1995. The most significant step was its inclusion in the Criminal Code. Nevertheless, it was Organic Law 3/2007 (2007) on Effective Equality between Women and Men that explicitly addressed sexual harassment and sex-based harassment. Article 7.1 defines sexual harassment as “any verbal or physical behavior of a sexual nature that has the purpose or effect of violating the dignity of a person, particularly when creating an intimidating, degrading, or offensive environment” (p. 12). Article 7.2 defines sex-based harassment as “any behavior carried out on the basis of a person’s sex, with the purpose or effect of violating their dignity and creating an intimidating, degrading, or offensive environment” (p. 12). Thus, the distinction between these two forms of harassment is clear: the former involves behaviors of a sexual nature, whereas the latter encompasses discriminatory situations primarily directed against women and individuals who challenge gender stereotypes (Bosch et al., 2012). Our study is grounded in the concepts established by current legislation. Organic Law 3/2007, in Article 48.1, imposes on employers the obligation to prevent sexual and sex-based harassment through appropriate working conditions and specific reporting procedures. This framework is reinforced by Royal Decree 901/2020, which requires the integration of such protocols into equality plans, mandatory for companies with 50 or more employees. In the case of Spanish universities, this requirement was strengthened following the enactment of Organic Law 2/2023, of March 22, on the University System (2023), which obliges governing councils to approve specific protocols and implement systematic measures for preventing and addressing violence, workplace harassment, and various forms of discrimination within the university setting, as stipulated in Article 46.l.

To deepen the conceptualization of sexual harassment as defined by legislation, this study adopts the theoretical framework of Fitzgerald et al. (1988, 1995), who conceptualize sexual harassment as a continuum of behaviors ranging from gender harassment and unwanted sexual attention to sexual coercion. Gender harassment refers to hostile or derogatory behaviors based on a person’s sex (e.g., sexist comments, degrading jokes, or humiliating treatment for being a woman or a man), aimed at reinforcing gender-based status hierarchies rather than expressing sexual desire. Unwanted sexual attention includes non-consensual sexual behaviors such as persistent invitations, sexualized comments about the body or appearance, or unwelcome touching or physical proximity. Finally, sexual coercion involves the use of pressure, blackmail, or threats (e.g., conditioning evaluations, grades, or work-related decisions on the acceptance of sexual favors) to obtain consent for sexual activities, constituting the most severe form of harassment. Accordingly, this study employs the Sexual Experiences Questionnaire (SEQ; Fitzgerald et al., 1995), which enables the identification of harassment based on the nature and intent of the behaviors. The scientific literature consistently recognizes the SEQ as one of the most widely used tools for assessing sexual harassment (Gutek et al., 2004).

In recent years, the evolution of digital communication has expanded the spectrum of behaviors that may constitute sexual or sex-based harassment. This has given rise to new manifestations known as technology-facilitated sexual violence (TFSV) (Powell & Henry, 2017; Ringrose et al., 2022). These behaviors include the sending of unwanted sexualized messages, the non-consensual dissemination of intimate images, online harassment with sexist or sexual content, and digital monitoring or control. Although these behaviors occur in virtual environments, they share with traditional forms of harassment the same structural foundations: gender inequality and the exercise of power over victims. Spanish legislation has also begun to recognize these digital forms of violence. Organic Law 10/2022 on the comprehensive guarantee of sexual freedom explicitly incorporates digital sexual violence, reinforcing the need to consider these behaviors within the broader conceptual framework of sexual and sex-based harassment. Consequently, TFSV can be understood as a contemporary extension of the continuum of behaviors described by Fitzgerald et al. (1988, 1995), now mediated by technology.

### 1.2. Prevalence of Sexual Harassment, Sex-Based Harassment, and TFSV in the University Context

The present study is situated within the analysis of sexual harassment, sex-based harassment, and TFSV in the university context; accordingly, we examine the situation both internationally and in Spain.

At the international level, several reports have raised concerns about the high incidence of sexual harassment and sex-based harassment in academic institutions (Lipinsky et al., 2022). In the North American context, the most recent findings indicate that between 27 and 30% of female students have experienced sexual harassment during their university studies (Sexual Assault and Related Harms Survey, University of Colorado Boulder, 2024; Cantor et al., 2024), and between 9 and 25% reported having suffered non-consensual sexual contact involving physical force. Additionally, 54.7% of female students reported receiving offensive sexual comments or jokes from members of the university community (Higher Education Sexual Misconduct and Awareness Survey, Cantor et al., 2024). Similarly, the study by DuBois and Pedneault (2025) showed that female university students face a 74% higher risk of sexual violence compared with non-university women of the same age.

In the European context, the magnitude of sexual harassment in higher education institutions was underscored in the UniSAFE project by Lipinsky et al. (2022), which found that 31% of students and staff reported having experienced sexual harassment at their institution. In the United Kingdom, the Office for Students (2025) reported that 24.5% of final-year undergraduate students had experienced sexual harassment during their studies, with women being nearly three times more likely than men to report such experiences (33 versus 12.2%). Moreover, 1.5% reported having engaged in sexual relations with academic staff, most often with lecturers who taught them.

Regarding TFSV, the international scientific literature highlights a high prevalence of TFSV behaviors among university students. Some studies estimate that 78.6% of university students have experienced at least one form of TFSV victimization (Yilmaz et al., 2025), with digital sexual harassment being particularly common (ranging from 53 to 70.4%), followed by gender-based harassment (43 to 58%), and image-based sexual abuse (30 to 34.1%) (Cripps & Stermac, 2018; Yilmaz et al., 2025; Walker et al., 2021).

Furthermore, research has documented a high level of student involvement in the exchange of sexually explicit content. Specifically, 71.10% of students reported having sent, and 78.26% having received, sexually explicit messages; 57.03% had sent, and 59.08% had received, explicit images; and 27.62% had sent, and 29.92% had received, explicit videos (Walker et al., 2021). Concerning perpetration and victimization, 16.37% of participants acknowledged sharing sexual images without consent, while 21.51% reported having been victimized (Walker et al., 2021).

Across studies, findings consistently indicate that women are more likely than men to experience TFSV victimization, particularly in cases involving digital sexual harassment, gender-based harassment, and the non-consensual distribution of sexually explicit messages, images, and/or videos (Cripps & Stermac, 2018; Walker et al., 2021).

In the Spanish context, and in comparison with the volume of international research, the number of reports and studies aimed at identifying the situation of sexual harassment and sex-based harassment in Spanish universities is notably lower. One pioneering report is the diagnostic study on sexual harassment and sex-based harassment conducted at the University of Vigo, Lameiras et al. (2018), which identified 35 students (30 women and 5 men), 13 faculty members (12 women and 1 man), and 9 administrative and service staff members (8 women and 1 man) as victims of sexual harassment. It also identified cases of sex-based harassment involving 37 female students, 17 female faculty members, and 4 female staff members (Lameiras et al., 2018). Similarly, the report from the Universidad Complutense de Madrid, Unidad de Igualdad de Género (2025) found that 6.5% of respondents had experienced sexual harassment within the university context; 26% reported having received sexual jokes, comments, or catcalls; 25% had been subjected to lewd gestures or looks; 4% had experienced unwanted touching; and 12% of women reported having been victims of sexist harassment at the university.

Beyond reports produced by the universities themselves, recent studies focusing on samples of Spanish university students also reveal clear trends regarding the distribution of victims and perpetrators, prevalence rates, and behaviors associated with sexual harassment and sex-based harassment. Regarding the profile of victims, studies consistently show that women are the main targets (fewer than 17% are men). Regarding the perpetrators’ profiles, 86% are identified as men (Hervías-Parejo, 2023). With respect to the prevalence of sexual harassment, findings indicate that between 8 and 23% of female university students in Spain have experienced some form of sexual harassment (Duque-Monsalve et al., 2024). The most frequent behaviors include sexualized comments, pressure and coercion, verbal violence, physical harassment, and technology-facilitated sexual harassment (Hervías-Parejo, 2023). Importantly, reporting rates remain extremely low, ranging from 2 to 7% (Duque-Monsalve et al., 2024).

### 1.3. Revictimization in Situations of Sexual Harassment, Sex-Based Harassment, TFSV

The different forms of sexual violence—such as sexual harassment, gender-based harassment, unwanted sexual attention, sexual coercion, and technology-facilitated sexual violence (TFSV)—are not isolated events but rather part of a continuum of the multiple forms of violence that women experience throughout their lives (Fitzgerald et al., 1995; Kelly, 1988). From this perspective, the theoretical framework of polyvictimization posits that women may be exposed to multiple forms of sexual violence across their lifespan, increasing the likelihood of repeated and overlapping victimization experiences (Breitenbecher, 2001). Within this framework, various studies have documented high rates of sexual revictimization (Relyea & Ullman, 2018; Regehr, 2022). The longitudinal study by Relyea and Ullman (2018) shows that nearly 50% of women who have survived some form of sexual violence are at heightened risk of experiencing new incidents, underscoring the repetitive nature of the phenomenon. Thus, previous experiences of sexual violence constitute one of the strongest predictors of different types of revictimization.

The scientific literature also demonstrates that revictimization extends into digital environments. Regehr’s (2022) study on TFSV shows that the non-consensual distribution of sexual content featuring the victim generates ongoing cycles of revictimization, in which each new viewing or redistribution of the material re-opens the trauma and prolongs the experience of violence. Technology-facilitated sexual victimization is associated both with prior offline sexual violence and with an increased likelihood of new victimization experiences in digital and in-person contexts, reinforcing the notion of the continuum of sexual violence (Regehr, 2022). Consistent with these findings, the systematic review by Patel and Roesch (2022) reports a high prevalence of TFSV among university students. Furthermore, Patel and Roesch (2022) reveal that women with prior experiences of offline sexual violence are more likely to suffer TFSV behaviors, reflecting consistent patterns of revictimization in digital settings.

### 1.4. The Present Study

This study seeks to address several gaps in the scientific literature on sexual harassment and sex-based harassment within the university context. As current evidence shows, most studies and reports, both internationally and in Spain, focus predominantly on university students, thereby relegating TRS to the background despite their central role within the institutional structure. This lack of scholarly attention has constrained our understanding of the risks, dynamics, and impacts that these forms of violence exert on individuals engaged in teaching, management, research, and supervision. Furthermore, the present study examines a novel, emerging, and still under-researched dimension in the literature: technology-facilitated sexual violence. This phenomenon has gained considerable relevance in increasingly digitalized university environments, yet it continues to receive limited academic scrutiny. The study is also situated within the framework of institutional protocols for the prevention of and response to sexual harassment in public universities. The integration of empirical research with institutional policy represents a meaningful contribution, as it provides evidence that can inform decision-making and guide the development of interventions tailored to both students and academic staff. For this reason, analyzing the scope of sexual harassment and sex-based harassment is crucial for understanding the magnitude and characteristics of the problem, as well as for identifying the predictors associated with these behaviors. Accordingly, the study pursued a dual aim: (a) to identify the prevalence of sexual harassment and sex-based harassment behaviors among TRS at a public university in northwestern Spain; and (b) to examine the predictive capacity of sociodemographic variables and prior experiences of harassment on the frequency of different forms of sexual victimization -gender harassment, unwanted sexual attention, and sexual coercion—within this institutional context, without inferring national prevalence. Comparative references to Spain and Europe are provided only for contextualization, not for generalization.

## 2. Methods

### 2.1. Population and Participants

The study employed a quantitative, cross-sectional, and descriptive-correlational design. The target population consisted of 1565 members of the TRS at a public university in northwestern Spain. This was a census-based study, as the entire target population was invited to participate, yielding 425 valid questionnaires, which corresponds to a response rate of 27.2%. This sample size is adequate for the purposes of the study (Bolarinwa, 2020; Vaghela, 2024) for the following reasons: first, sample size estimation using Cochran’s formula indicates that, assuming a 95% confidence level and maximum population variability, a sample of approximately 385 cases is typically sufficient. Thus, the 425 responses obtained exceed this threshold. Second, with 425 respondents, the margin of error at a 95% confidence level is approximately ±4%, which is considered highly satisfactory. Although the census invitation covered the full TRS population at the institution, response patterns by professional rank and disciplinary area were not fully balanced; therefore, the sample is not representative of the Spanish university system nor of all staff at the institution, and estimates should be read as institution-specific.

The sample consisted of 425 respondents, of whom 48.9% (*n* = 208) were women, 50.6% (*n* = 215) were men, and 0.5% (*n* = 2) identified as “not identified”. All participants were affiliated with a public university in northwestern Spain. The mean age was 45.88 years (*SD* = 22.2), ranging from 22 to 68 years.

Regarding professional category, 40% held full-time permanent positions, 38.4% were full-time with non-permanent contracts, and 21.6% were part-time with non-permanent contracts. Concerning the scientific-academic field, 29.4% belonged to the legal and social sciences, 25.6% to the natural sciences, 23.8% to engineering and architecture, 16.7% to the arts and humanities, and 4.5% to health sciences. This sample is not representative of the professional category or the scientific field.

### 2.2. Measures

For this study, we used a questionnaire comprising the following items and scales:

*Demographic questions:* sex (male, female, not identified); age; Professional category (TRS with permanent appointments, TRS with full-time non-permanent contracts, and TRS with part-time non-permanent contracts); and scientific-academic field (legal and social sciences, natural sciences, engineering and architecture, arts and humanities, and health sciences).

*Sexual harassment and sex-based harassment behaviors:* To assess the incidence of sexual harassment and sex-based harassment experiences, two direct questions were posed to participants: whether they had ever experienced a situation of sexual harassment or sex-based harassment at the university, using a dichotomous response format (yes/no). These questions were designed to capture respondents’ explicit acknowledgment of the phenomenon, in other words, their self-identification as victims based on their lived experience and in alignment with the institutional and regulatory framework that defines both forms of harassment. In this way, the variables reflect the subjectively recognized presence of such behaviors, thereby complementing other, more behavioral or indirect measures of the phenomenon.

*Identification of sexual experiences:* The Sexual Experiences Questionnaire (SEQ) by Fitzgerald et al. (1988) was used in its Spanish adaptation by Lameiras et al. (2018). This 19-item instrument assesses the frequency with which respondents have allegedly experienced these behaviors while employed by the defendant organization. The SEQ is composed of three subscales: (i) Gender Harassment (seven item; e.g., “told suggestive stories or offensive jokes”) refers to sexist remarks and behaviors that convey insulting, hostile, and degrading attitudes about women that are not necessarily aimed at sexual cooperation; (ii) Unwanted Sexual Attention (five items; e.g., “made unwanted attempts to stroke or fondle you”) refers to offensive and unwelcome verbal and nonverbal sexual behavior, including forceful attempts to stroke, fondle, or touch, as well as attempted or completed forced sex; and (iii) Sexual Coercion (seven items; e.g., “implied faster promotions or better treatment if you were sexually cooperative”). The extortion of sexual cooperation in return for job-related considerations is referred to as sexual coercion. In this study, the SEQ subscales demonstrated adequate reliability. Responses were provided on a 5-point Likert-type scale, ranging from 0 (*never*) to 4 (*many times*). The Gender Harassment subscale yielded an α = 0.80, the Unwanted Sexual Attention subscale an α = 0.88, and the Sexual Coercion subscale an α = 0.97.

*Technology-facilitated sexual harassment behaviors:* A three-item scale adapted from Ringrose et al. (2022) was employed to assess the frequency of sexually offensive behaviors occurring through digital media within the university context (e.g., “Made unwanted sexual comments via digital media; made sexist remarks about your body through digital media; sent an unsolicited explicit image of an intimate body part such as the penis or breasts”). Responses were recorded on a 5-point Likert scale ranging from 1 (*never*) to 5 (*very often*). In this study, the alpha was 0.80.

*Awareness of sexual harassment and sex-based harassment cases* (ad hoc): Participants were asked whether they were aware of any case of sexual harassment or sex-based harassment that had occurred at the university involving another person, using a dichotomous response format (yes/no).

*Awareness of the existence of the university’s sexual harassment and sex-based harassment protocol* (ad hoc): A closed-ended question was posed with a dichotomous response format (yes/no).

*Perception of the effectiveness of the university’s sexual harassment and sex-based harassment protocol* (ad hoc): Participants were asked to rate the perceived effectiveness of the protocol using a 4-point Likert scale ranging from 1 (*not effective at all*) to 4 (*very effective*).

### 2.3. Procedure

An online questionnaire was created using the university’s digital platform in collaboration with the Quality Assurance Service. At the beginning of the questionnaire, participants were informed about the purpose of the study and provided with definitions of sexual harassment and sex-based harassment as established in Law 3/2007. Confidentiality of the data was guaranteed, and informed consent was obtained prior to participation.

The fieldwork began with a dissemination campaign through an official communication on the university’s social media channels and via email to the TRS community distribution list. This message outlined the research objectives and emphasized the importance and necessity of TRS participation. Over a two-month period, the Quality Assurance Service sent individualized emails to TRS members inviting them to complete the online questionnaire. The estimated time to complete the questionnaire was approximately 15–20 min.

### 2.4. Statistical Analysis

The following analyses were performed using IBM SPSS v.24 software. Firstly, we calculated descriptive statistics, chi-square tests, frequencies, and mean differences by gender for the variables and scales using Student’s *t*-test. Secondly, we computed bivariate correlations between the scales/subscales and the variables. Thirdly, before conducting the hierarchical regression analyses, we examined the contingency tables to identify any empty or sparsely populated cells in the combinations of categorical variables included in the models. This inspection revealed no empty cells or issues of complete separation that would compromise parameter estimation. Nonetheless, given the low mean scores observed in the SEQ subscales, we acknowledge that statistical power to detect small effects may be limited, and that the resulting estimates should therefore be interpreted with appropriate caution. Subsequently, we conducted a three-step hierarchical linear regression to predict sexual harassment experiences among TRS members, entering gender harassment, unwanted sexual attention, and sexual coercion as predictors.

## 3. Results

### Descriptive Statistics

Firstly, we found that 7.2% (*n* = 15) of female TRS explicitly self-identified as victims of sexual harassment (χ^2^_(1)_ = 13.21, *p* = 0.001) and 14.2% (*n* = 30) reported having experienced gender-based harassment within the university (χ^2^_(1)_ = 30.32, *p* = 0.001), whereas male TRS reported only one self-identified case in each category (see Table 1).

Female TRS also demonstrated greater awareness of cases of sexual harassment (20.7%, *n* = 43) and gender-based harassment (18.8%, *n* = 39) compared to their male counterparts (13.5%, *n* = 29 and 9.8%, *n* = 21, respectively), with significant differences in both instances (χ^2^_(1)_ = 3.86, *p* = 0.05; χ^2^_(1)_ = 7.01, *p* = 0.01). However, no significant differences emerged regarding knowledge of the university’s sexual harassment protocol; both male and female TRS exhibited similar levels of awareness (47.4% and 50.2%, respectively).

Secondly, we compared the mean differences across the SEQ subscales by sex (see Table 2). Overall, the analyses revealed low mean scores, indicating a generally low frequency of behaviors across all three SEQ subscales. Despite this low base rate, female TRS exhibited significantly higher levels of gender harassment (*t* = 4.72, *p* = 0.001) and unwanted sexual attention (*t* = 3.45, *p* = 0.001) than their male counterparts. Furthermore, female TRS reported higher levels of technology-facilitated sexual harassment compared to male TRS, with significant differences (*t* = 2.45, *p* = 0.046).

Table 3 presents the correlation coefficients between the analyzed variables and subscales. The three SEQ subscales exhibited strong positive intercorrelations. Notably, the Technology-Facilitated Sexual Harassment scale showed robust correlations with all three SEQ subscales: Gender Harassment (*r* = 0.41, *p* < 0.01), Unwanted Sexual Attention (*r* = 0.80, *p* < 0.01), and Sexual Coercion (*r* = 0.86, *p* < 0.01). Furthermore, the three SEQ subscales were also positively associated with being a victim of sexual harassment and gender-based harassment.

TRS members who had experienced more instances of gender harassment (*r* = 0.22, *p* < 0.01) and unwanted sexual attention (*r* = 0.13, *p* < 0.01) tended to have greater awareness of other cases of sexual harassment within the university. Similarly, TRS members who had experienced higher levels of gender harassment (*r* = 0.34, *p* < 0.01), unwanted sexual attention (*r* = 0.21, *p* < 0.01), and sexual coercion (*r* = 0.15, *p* < 0.01) were more likely to be aware of other cases of gender-based harassment at the university. Conversely, TRS members who had experienced more situations of gender harassment (*r* = −0.24, *p* < 0.01) and unwanted sexual attention (*r* = −0.12, *p* < 0.01) perceived the university’s sexual harassment protocol as ineffective.

Female TRS experienced gender harassment (*r* = −0.23, *p* < 0.01), unwanted sexual attention (*r* = −0.17, *p* < 0.01), and technology-facilitated sexual harassment (*r* = −0.12, *p* < 0.01) more frequently. They also identified themselves as victims of sexual harassment (*r* = −0.17, *p* < 0.01) and gender-based harassment (*r* = −0.26, *p* < 0.01), and reported greater awareness of other cases of sexual harassment (*r* = −0.10, *p* < 0.05) and gender-based harassment (*r* = −0.13, *p* < 0.01) within the university.

Next, the regression model was tested using hierarchical multiple regression to assess the predictive strength of the variables—sex, age, professional category, scientific-academic field, victimization by sexual harassment, victimization by gender-based harassment, technology-facilitated sexual harassment behaviors, awareness of cases of sexual and gender-based harassment, knowledge of the existence of the university’s sexual harassment and gender-based harassment protocol, and perception of the protocol’s effectiveness—in relation to the frequency of experienced sexual behaviors (SEQ subscales as dependent variables [DV]) (see Table 4, Table 5 and Table 6). Although the regression models met the minimum variability requirements for the included variables, the low prevalence of certain behaviors suggests that some coefficients may be sensitive to small fluctuations in the sample. Consequently, the results should be interpreted as exploratory estimates.

Table 4 presents the results of the hierarchical multiple regression analysis assessing the predictive strength of the study variables for the frequency of gender harassment experiences (DV). The four variables were entered into Step 1 of the analysis, accounting for a significant 6.7% of the variance. In Step 2, the seven predictors were entered into the regression analysis, which explained 40% of the variance in the model. When the predictor variables were added, they contributed an additional 33.7% to the variance, specifically in the frequency of experiencing gender harassment situations, Δ*R*^2^ = 0.337, *F*(11, 308) = 20.8, *p* < 0.001. In the final model (Step 3), several variables showed significant effects on the frequency of gender-harassment experiences. Being a woman exhibited a marginal effect (β = 0.09, *t* = −1.84, *p* = 0.05). Having previously experienced sex-based harassment emerged as the strongest predictor (β = 0.32, *t* = 5.98, *p* = 0.001), followed by technology-facilitated sexual harassment behaviors (β = 0.26, *t* = 4.33, *p* = 0.001). Awareness of cases of sex-based harassment was also positively associated with the frequency of such experiences (β = 0.13, *t* = 2.89, *p* = 0.004). Finally, perceived effectiveness of the institutional protocol had a significant negative effect (β = –0.14, *t* = –3.32, *p* = 0.001), indicating that higher evaluations of the protocol were associated with lower levels of reported harassment.

Table 5 presents the results of the hierarchical multiple regression analysis assessing the predictive strength of the study variables for the frequency of unwanted sexual attention experiences (DV). The four variables were entered at Step 1 of the analysis, accounting for a significant 3.2% of the variance. In Step 2, the seven predictors were entered into the regression analysis, which explained a total of 64.2% of the variance in the model. When the predictor variables were added, they contributed an additional 61% of the variance, specifically in the frequency of experiencing unwanted sexual attention, Δ*R*^2^ = 0.610, *F*(11, 308) = 55.10, *p* < 0.001. In the final model (Step 3), four predictors had significant effects on the frequency of unwanted sexual attention experiences. Having been a victim of sexual harassment was positively associated with this frequency (β = 0.23, *t* = 6.18, *p* = 0.001), as was having experienced sex-based harassment (β = 0.15, *t* = 3.49, *p* = 0.001). Sexual coercion emerged as the strongest predictor in the model (β = 0.31, *t* = 7.03, *p* = 0.001), followed by technology-facilitated sexual harassment behaviors, which also showed a strong and significant effect (β = 0.30, *t* = 6.60, *p* = 0.001). These results indicate that prior victimization experiences and various forms of sexual harassment, occurring both in person and through digital means, contribute substantially to the frequency of unwanted sexual attention reported.

Table 6 presents the results of the hierarchical multiple regression analysis assessing the predictive strength of the study variables for the frequency of sexual coercion experiences (DV). The four variables were entered at Step 1 of the analysis, accounting for a significant 1.4% of the variance. In Step 2, the seven predictors were entered into the regression analysis, which explained 54% of the variance in the model. When the predictor variables were added, they contributed an additional 52% of the variance, specifically in the frequency of sexual coercion experiences, Δ*R*^2^ = 0.526, *F*(11, 338) = 36.07, *p* < 0.001. In the final model (Step 3), three predictors had significant effects on the frequency of sexual coercion experiences. Professional category exhibited a significant negative effect (β = –0.08, *t* = −2.09, *p* = 0.037), indicating that higher professional ranks are associated with lower frequencies of sexual coercion. Unwanted sexual attention emerged as the strongest predictor in the model (β = 0.41, *t* = 7.96, *p* = 0.001), followed by technology-facilitated sexual harassment behaviors (β = 0.40, *t* = 7.03, *p* = 0.001), both of which were positively associated with the frequency of sexual coercion. These results indicate that prior victimization and digitally mediated forms of sexual harassment constitute key factors in explaining variability in sexual coercion experiences.

## 4. Discussion

In the present study, we observed that sexual and sex-based harassment continue to be present in this public university in northern Spain. Notably, several female TRS explicitly reported having felt like victims of sexual harassment and sex-based harassment. In addition, they were the group most likely to report experiences of gender harassment, unwanted sexual attention, and technology-facilitated sexual violence when compared with their male colleagues. These findings are consistent with both national and international research showing that women are the primary targets of sexual and sex-based harassment in both offline and online contexts (Johnson & Bennett, 2015; Finchilescu & Dugard, 2018; Lameiras et al., 2018; Muhonen, 2016; Universidad Complutense de Madrid, Unidad de Igualdad de Género, 2018; Mills et al., 2025).

An interesting finding of this study is that female TRS of this university who experienced situations of gender harassment, unwanted sexual attention, and sexual coercion reported knowing about more cases of other victims of sexual and sex-based harassment within the university than their male colleagues. One factor contributing to this situation, as evidenced by Evans et al. (2019), is that women in academic positions frequently receive confidences and accounts of cases from colleagues and students, reinforcing their role as knowledge holders through informal support networks. In the same vein, Ludwig et al. (2024) note that women in universities—both faculty and students—tend to report more experiences of discrimination and sexual harassment. This pattern of greater exposure among women, combined with the trust victims place in them, allows female professors and researchers to accumulate greater visibility and awareness of cases within the university environment.

Another important finding is that fewer than half of this university’s TRS members are aware of the institution’s official protocol on sexual and sex-based harassment. Currently, Spanish universities are legally required to have a harassment protocol in place (Royal Decree 901/2020). However, female TRS who have self-identified as victims of sexual harassment behaviors reported, such as unwanted sexual attention or gender harassment, consider these protocols ineffective in combating such forms of sexual violence. Studies conducted at European and Spanish universities indicate that the formal presence of protocols does not guarantee that teaching and research staff are aware of them or feel supported by them, highlighting issues of dissemination, lack of specific training, and low confidence in institutional channels (Lombardo & Bustelo, 2021).

Moreover, our focus was on determining which study variables predict the frequency of sexual experiences suffered (SEQ), manifested as gender harassment, unwanted sexual attention, and sexual coercion. In interpreting these findings, it is important to acknowledge that, although the regression models met the minimum variability requirements for the included variables, the overall low prevalence of several behaviors introduces certain limitations. Specifically, this reduced base rate may render some coefficients sensitive to minor fluctuations in the sample. As a result, the patterns identified should be viewed as exploratory and interpreted with caution, rather than as definitive estimates of population-level effects. The results of the hierarchical regression models confirm the usefulness of Fitzgerald et al.’s (1995) three-dimensional framework for understanding the dynamics of sexual harassment, showing that gender harassment, unwanted sexual attention, and sexual coercion constitute interrelated yet distinct dimensions within a continuum of victimization (Fitzgerald et al., 1995; Gutek et al., 2004). The fact that, across all three regression models, prior victimization experiences and technology-facilitated harassment behaviors emerged as robust predictors suggests that the mechanisms underlying the risk of sexual revictimization—such as cumulative vulnerability and the normalization of hostile environments—are also present in the university and workplace contexts examined in this study (Messman-Moore & Long, 2003; Relyea & Ullman, 2018). Thus, women with elevated rates of revictimization tend to have experienced previous forms of sexual violence. Furthermore, this pattern aligns with studies highlighting the influence of situational and relational variables, such as asymmetric power dynamics or permissive organizational climates, on the recurrence of sexual harassment experiences (Relyea & Ullman, 2018; Zara et al., 2025). These findings therefore underscore the need to conceptualize sexual harassment not as an isolated event but as a series of cumulative processes that mutually reinforce one another across different forms of harassment, primarily affecting women, both in face-to-face and digital settings (National Academies of Sciences, Engineering, and Medicine, 2018; Powell & Henry, 2017).

In the context of gender harassment, having been a victim of sex-based harassment emerged as the strongest predictor in the hierarchical regression model. This finding is consistent with the conceptualization of gender-based hostility as a devaluative form of harassment aimed at putting individuals “in their place” when they challenge or deviate from gender stereotypes (Fitzgerald et al., 1995; Leskinen et al., 2011). Numerous studies have demonstrated that prior exposure to degrading comments, sexist jokes, or dismissive treatment on the basis of gender increases the likelihood of experiencing additional forms of harassment, both in university and workplace settings (Hill & Silva, 2005; Ilies et al., 2003). In this regard, the positive association between awareness of cases of sex-based harassment and the frequency of personal experiences can be interpreted in two ways: on the one hand, as an indicator that such behaviors are frequent in the university context; and on the other, as a reflection of greater sensitivity and recognition capacity among members of the academic community (Cortina & Berdahl, 2008; National Academies of Sciences, Engineering, and Medicine, 2018). Likewise, the negative association between perceived effectiveness of the institutional protocol and the frequency of harassment reinforces existing evidence linking normative clarity, procedural credibility, and effective sanctioning to lower levels of sexual harassment within organizations (Holland et al., 2016; Willness et al., 2007). This result also aligns with recent findings in the field of digital sexual violence, which show that strong ethical norms in online environments and clearly defined regulatory frameworks function as protective factors by reducing the likelihood of perpetration or tolerance of abusive behaviors, an interpretation that may extend to perceptions of institutional protocol effectiveness (Powell & Henry, 2017; Vitis & Gilmour, 2017).

In relation to the results of the regression model for unwanted sexual attention, the finding that sexual coercion and technology-facilitated sexual harassment emerged as the strongest predictors suggests that unwanted advances occupy an intermediate position within the escalation of sexual violence (Fitzgerald et al., 1995; McDonald, 2012). Unwanted sexual attention, understood as a form of “come-on,” can evolve into more explicit forms of sexual coercion in contexts marked by power asymmetries and sexist norms, particularly when clear boundaries are not established or when institutional environments downplay its severity (Fitzgerald et al., 1995; Leskinen et al., 2011). Similarly, research on online sexual harassment indicates that its manifestations, such as persistent sexualized messaging, the non-consensual sending of sexual content, or pressure to exchange intimate material, intertwine with face-to-face interactions and amplify victims’ exposure and vulnerability, especially among university and young adult populations (Powell & Henry, 2017; Reed et al., 2016). The strong association observed between prior experiences of sexual harassment and unwanted sexual attention is consistent with revictimization models demonstrating that exposure to initial incidents heightens vulnerability to subsequent forms of sexual violence, both in intimate relationships and in other settings (Classen et al., 2005; Zara et al., 2025). Taken together, these results underscore the importance of early interventions aimed at interrupting the progression from unwanted sexual attention to sexual coercion, incorporating both offline and online dimensions (Powell & Henry, 2017).

Finally, in the hierarchical regression model predicting the frequency of sexual coercion, the protective effect associated with higher professional ranks suggests that occupying senior hierarchical positions and having greater employment stability reduces the risk of experiencing more severe forms of harassment. Several studies have shown that individuals in precarious employment conditions, young women, and students face greater vulnerability, as they occupy positions with limited structural power and are therefore more exposed to coercive dynamics (Hill & Silva, 2005; McDonald, 2012; Shupe et al., 2002). Sexual coercion has been described as the most severe dimension of harassment, closely linked to power dynamics, control, and fear of retaliation, which helps explain its higher prevalence among individuals situated in subordinate hierarchical positions (Fitzgerald et al., 1995; McDonald, 2012). At the same time, the finding that unwanted sexual attention and technology-facilitated sexual harassment are the strongest predictors of sexual coercion supports the hypothesis of a continuum in which behaviors initially perceived as normalized or ambiguous may escalate into direct pressure to obtain sexual favors (Fitzgerald et al., 1995; Powell & Henry, 2017). Consistent with this perspective, recent research on digital sexual violence indicates that coercion through threats of disseminating intimate images, revealing personal data to facilitate physical tracking, or blackmail constitute specific forms of technologically mediated coercion that are particularly prevalent among young people, sexual minorities, and women (Powell & Henry, 2017; Reed et al., 2016). Taken together, these results demonstrate that sexual coercion cannot be understood in isolation but rather as an advanced stage within a pattern of repeated victimization that integrates both prior offline harassment and online forms of abuse.

### Limitations and Future Directions

This study presents several limitations that should be considered. First, although the results obtained allow us to identify patterns consistent with the national and international literature on sexual and sex-based harassment in university settings, these findings cannot be directly extrapolated to other universities or to the Spanish higher education system as a whole. The study is based on data from a single public university in northern Spain, and participation was voluntary and non-random, limiting the external representativeness of the sample. The distribution of respondents across scientific–academic fields and professional categories is also uneven, which may influence perceptions and the reported prevalence of harassment in certain disciplinary areas.

Additionally, the exclusive use of an online questionnaire may have reduced participation among individuals with limited time availability or lower levels of digital proficiency, and the self-administered format may introduce variation in the interpretation of some items or potential social desirability bias. As a cross sectional, self-report study, the findings reflect perceptions at a single point in time and do not permit the establishment of causal relationships.

Another limitation is that some of the forms of victimization examined represent low incidence behaviors within the sample. Although the regression models satisfied the mini- mum variability requirements, the low prevalence of certain experiences means that some coefficients may be sensitive to small sample fluctuations and should therefore be interpreted as exploratory rather than as stable estimates. Nevertheless, the consistency between our results and patterns documented in previous research -such as women’s greater exposure to harassment, the relevance of technology facilitated forms of victimization, and the association between prior victimization and increased vulnerability, suggests that the dynamics observed in this institution are not isolated, but align with trends described in other university contexts.

Future studies should incorporate larger and, ideally, multi-institutional samples to increase statistical precision and enable comparisons across institutional cultures. Expanding the geographical scope to include other Spanish and European universities would contribute to a more comprehensive understanding of the phenomenon. Incorporating an intersectional perspective, considering variables such as sexual orientation, ethnicity, religion, disability, and other sociodemographic factors, would also deepen the analysis. Moreover, examining in greater detail the role of technology facilitated harassment and its relationship with revictimization, as well as complementing quantitative approaches with qualitative methodologies such as in-depth interviews, particularly with victims, would provide richer insights into experiences, reporting barriers, and institutional responses.

## 5. Conclusions

This study indicates that sexual and sex-based harassment continues to be present in this public university in northern Spain, with female professors and researchers being the group most affected. Within this institutional context, women reported a higher frequency of behaviors such as gender harassment, unwanted sexual attention, and technology-facilitated sexual harassment. Furthermore, the limited awareness among TRS members of the university’s official protocol for addressing sexual and sex-based harassment—together with the perception expressed by some victimized women that existing mechanisms are insufficient or ineffective—points to persistent institutional challenges that warrant attention and improvement within this specific university setting. Although Spanish universities are legally required to implement anti-harassment protocols, these findings suggest that formal compliance alone is insufficient. Without adequate dissemination, training, and trust in institutional procedures, such protocols cannot function as effective protective tools for academic staff.

Finally, the hierarchical regression models in this study provide relevant implications for institutional prevention and intervention strategies within this public university in northern Spain. The consistent emergence of technology-facilitated sexual harassment as a strong predictor across all three dimensions examined aligns with research that conceptualizes TFSV as a cross-cutting phenomenon that permeates, intensifies, and amplifies traditional forms of harassment (Powell & Henry, 2017; Vitis & Gilmour, 2017). Consequently, university protocols should explicitly incorporate digital sexual violence both in the definition of sanctionable behaviors and in the pathways for reporting, protection, and support—as recommended by recent reviews focusing on young populations and educational environments (Reed et al., 2016).

Moreover, the relevance of prior victimization underscores the need for targeted programs that extend beyond case-by-case management. This approach requires providing ongoing follow-up for affected individuals and addressing, in a preventive manner, the factors associated with revictimization (Classen et al., 2005; Relyea & Ullman, 2015). Finally, the results point to the need for comprehensive strategies that simultaneously address gender harassment, unwanted sexual attention, and sexual coercion, both offline and online (National Academies of Sciences, Engineering, and Medicine, 2018; Powell & Henry, 2017). Preventing sexual harassment in this university requires not only formal mechanisms but also broader cultural and structural transformations that challenge gender-based power imbalances and promote safe, equitable, and respectful academic environments.

## Figures and Tables

**Table 1:** Frequency distribution and Chi-Square test (χ^2^) results for female and male TRS.

	Total	Female TRS	Male TRS
Have you ever experienced sexual harassment at the university?			
Yes	3.8% (*n* = 16)	7.2% (*n* = 15)	0.5% (*n* = 1)
No	96.2% (*n* = 407)	92.8% (*n* = 193)	99.5% (*n* = 214)
	χ^2^_(1)_ = 13.21; *p* = 0.001		
Have you ever experienced sex-based harassment at the university?			
Yes	7.3% (*n* = 31)	14.4% (*n* = 30)	0.5% (*n* = 1)
No	92.7% (*n* = 392)	85.6% (*n* = 178)	99.5% (*n* = 214)
	χ^2^_(1)_ = 30.32, *p* = 0.001		
Do you know anyone who has experienced sexual harassment at the university?			
Yes	17% (*n* = 72)	20.7% (*n* = 43)	13.5% (*n* = 29)
No	83% (*n* = 351)	79.3% (*n* = 165)	86.5% (*n* = 186)
	χ^2^_(1)_ = 3.86, *p* = 0.05		
Do you know anyone who has experienced sex-based harassment at the university?			
Yes	14.2% (*n* = 60)	18.8% (*n* = 39)	9.8% (*n* = 21)
No	85.8% (*n* = 363)	81.3% (*n* = 169)	90.02% (*n* = 194)
	χ^2^_(1)_ = 7.01, *p* = 0.01		
Are you familiar with the university’s harassment protocol?			
Yes	48.8% (*n* = 209)	50.2 % (*n* = 110)	47.4% (*n* = 99)
No	51.2% (*n* = 180)	49.8% (*n* = 80)	52.6% (*n* = 100)
	χ^2^_(1)_ = 2.80, *p* = 0.246		

Note: TRS: teaching and research staff.

**Table 2:** Differences of means in SEQ subscales, Technology-facilitated sexual harassment and perceived effectiveness of the protocol by female and male TRS.

	*M* (*SD*)		Student-t	*p*
	Female TRS	Male TRS		
SEQ (Subscales) Gender Harassment	1.45 (0.64)	1.21 (0.32)	4.72	0.001
Unwanted Sexual Attention	1.14 (0.49)	1.02 (0.13)	3.45	0.001
Sexual Coercion	1.04 (0.30)	1.00 (0.01)	1.64	0.103
Technology-Facilitated Sexual Harassment	1.08 (0.36)	1.01 (0.07)	2.42	0.016
Perceived effectiveness of the protocol	2.64 (0.75)	2.58 (0.84)	0.730	0.466

Note: M: mean; SD (standard deviation). TRS: teaching and research staff.

**Table 3:** Pearson correlations among study variables and SEQ subscales.

	Gender Harassment	Unwanted Sexual Attention	Sexual Coercion
Sex	−0.23 **	−0.17 **	−0.08
Age	−0.007	0.03	0.03
Professional category	−0.09	−0.11 *	−0.06
Scientific-academic field	−0.01	−0.01	−0.03
Victim of sexual harassment	0.27 **	0.51 **	0.36 **
Victim of sex-based harassment	0.50 **	0.41 **	0.25 **
Awareness of cases of sexual harassment	0.22 **	0.13 **	0.04
Awareness of cases of sex-based harassment	0.34 **	0.21 **	0.15 **
Technology-facilitated sexual harassment behaviors	0.41 **	0.80 **	0.86 **
Knowledge of the protocol	0.06	−0.09	−0.13 **
Perceived effectiveness of the protocol	−0.24 **	−0.12 *	−0.06
Gender harassment	--	0.44 **	0.32 **
Unwanted sexual attention	--	--	0.73 **

Note: ** *p* < 0.01. * *p* < 0.05.

**Table 4:** Hierarchical multiple regression analysis predicting the frequency of gender harassment experiences.

	B	SE B	β	*t* (*p*)
Step 1				
Sex	−0.25	0.05	−0.24	−4.64 (0.001)
Age	0.000	0.001	−0.01	−0.21 (0.828)
Professional category	−0.04	0.02	−0.09	−1.75 (0.079)
Scientific-academic field	0.003	0.01	0.007	0.14 (0.889)
*F*(df., df error)			6.17 *** (4, 345)	
*R*^2^			0.067	
Step 2				
Sex	−0.08	0.04	−0.08	−1.88 (0.050)
Age	0.000	0.001	−0.02	−0.45 (0.653)
Professional category	−0.02	0.01	−0.06	−1.50 (0.133)
Scientific-academic field	−0.01	0.01	−0.03	−0.88 (0.375)
Victim of sexual harassment	−0.02	0.13	−0.01	−0.18 (0.851)
Victim of sex-based harassment	0.64	0.10	0.33	6.22 (0.001)
Awareness of cases of sexual harassment	0.10	0.06	0.07	1.73 (0.084)
Awareness of cases of sex-based harassment	0.22	0.06	0.15	3.23 (0.001)
Unwanted sexual attention	0.07	0.13	0.04	0.58 (0.562)
Sexual coercion	−0.28	0.35	−0.05	−0.81 (0.418)
Technology-facilitated sexual harassment behaviors	0.94	0.20	0.28	4.67 (0.001)
*F*(df., df error)			20.8 *** (11, 338)	
*R*^2^			0.404	
Δ*R*^2^			0.337	
Δ*F*^2^			27.3 (0.001)	
Step 3				
Sex	−0.09	0.04	−0.09	−1.84 (0.049)
Age	−0.001	0.001	−0.02	−0.56 (0.572)
Professional category	−0.02	0.01	−0.05	−1.33 (0.182)
Scientific-academic field	−0.01	0.01	−0.04	−0.98 (0.323)
Victim of sexual harassment	−0.06	0.13	−0.02	−0.44 (0.659)
Victim of sex-based harassment	0.61	0.10	0.32	5.98 (0.001)
Awareness of cases of sexual harassment	0.08	0.06	0.06	1.43 (0.153)
Awareness of cases of sex-based harassment	0.20	0.06	0.13	2.89 (0.004)
Unwanted sexual attention	0.10	0.13	0.05	0.77 (0.437)
Sexual coercion	−0.26	0.35	−0.04	−0.74 (0.456)
Technology-facilitated sexual harassment behaviors	0.87	0.20	0.26	4.33 (0.001)
Knowledge of the protocol	0.01	0.04	0.01	0.26 (0.793)
Perceived effectiveness of the protocol	−0.09	0.02	−0.14	−3.32 (0.001)
*F*(df., df error)			17.8 *** (14, 335)	
*R*^2^			0.427	
Δ*R*^2^			0.023	
Δ*F*^2^			4.57 (0.004)	

Note: *** *p* < 0.001.

**Table 5:** Hierarchical multiple regression analysis predicting the frequency of unwanted sexual attention experiences.

	B	SE B	β	*t* (*p*)
Step 1				
Sex	−0.09	0.03	−0.17	−3.26 (0.001)
Age	0.001	0.001	0.01	0.17 (0.858)
Professional category	−0.01	0.01	−0.04	−0.87 (0.385)
Scientific-academic field	0.004	0.01	0.02	0.41 (0.676)
*F*(df., df error)			2.8 * (4, 345)	
*R*^2^			0.032	
Step 2				
Sex	−0.008	0.01	−0.01	−0.40 (0.685)
Age	0.001	0.001	−0.01	−0.35 (0.720)
Professional category	−0.01	0.008	−0.05	−1.56 (0.117)
Scientific-academic field	−0.002	0.006	−0.009	−0.27 (0.783)
Victim of sexual harassment	0.33	0.05	0.23	6.19 (0.001)
Victim of sex-based harassment	0.15	0.04	0.15	3.63 (0.001)
Awareness of cases of sexual harassment	0.04	0.02	0.05	1.63 (0.104)
Awareness of cases of sex-based harassment	0.001	0.02	0.001	0.03 (0.973)
Sexual Coercion	0.97	0.13	0.31	7.13 (0.001)
Technology-facilitated sexual harassment behaviors	0.53	0.08	0.30	6.62 (0.001)
Gender Harassment	0.01	0.02	0.02	0.58 (0.562)
*F*(df., df error)			55.0 *** (11, 338)	
*R*^2^			0.642	
Δ*R*^2^			0.610	
Δ*F*^2^			82.1 (0.001)	
Step 3				
Sex	−0.011	0.01	−0.02	−0.58 (0.556)
Age	−7.59	0.001	−0.007	−0.19 (0.849)
Professional category	−0.01	0.008	−0.05	−1.70 (0.090)
Scientific-academic field	−0.001	0.006	−0.006	−0.19 (0.846)
Victim of sexual harassment	0.33	0.05	0.23	6.18 (0.001)
Victim of sex-based harassment	0.15	0.04	0.15	3.49 (0.001)
Awareness of cases of sexual harassment	0.04	0.02	0.06	1.92 (0.055)
Awareness of cases of sex-based harassment	0.008	0.02	0.01	0.28 (0.780)
Sexual Coercion	0.95	0.13	0.31	7.03 (0.001)
Technology-facilitated sexual harassment behaviors	0.53	0.08	0.30	6.60 (0.001)
Gender Harassment	0.01	0.02	0.03	0.77 (0.437)
Knowledge of the protocol	−0.02	0.02	−0.04	−1.315 (0.189)
Perceived effectiveness of the protocol	0.01	0.01	0.03	0.88 (0.378)
*F*(df., df error)			43.5 *** (14, 335)	
*R*^2^			0.645	
Δ*R*^2^			0.004	
Δ*F*^2^			1.15 (0.328)	

Note: *** *p* < 0.001; * *p* < 0.05.

**Table 6:** Hierarchical multiple regression analysis predicting the frequency of sexual coercion experiences.

	B	SE B	β	*t* (*p*)
Step 1				
Sex	−0.01	0.01	−0.08	−1.53 (0.127)
Age	−0.001	0.001	−0.03	0.57 (0.568)
Professional category	0.006	0.004	0.08	1.59 (0.112)
Scientific-academic field	0.001	0.003	0.01	0.21 (0.833)
*F*(df., df error)			1.24 (4, 345)	
*R*^2^			0.014	
Step 2				
Sex	0.003	0.007	0.01	0.44 (0.659)
Age	6.71	0.001	0.01	0.45 (649)
Professional category	−0.006	0.003	−0.08	−2.11 (0.035)
Scientific-academic field	0.001	0.002	−0.005	−0.13 (0.890)
Victim of sexual harassment	0.01	0.02	0.03	0.74 (0.457)
Victim of sex-based harassment	−0.01	0.01	−0.05	−1.03 (0.300)
Awareness of cases of sexual harassment	0.007	0.009	0.03	0.79 (0.425)
Awareness of cases of sex-based harassment	−0.005	0.01	−0.01	−0.44 (0.655)
Technology-facilitated sexual harassment behaviors	0.23	0.02	0.41	7.99 (0.001)
Gender Harassment	−0.007	0.008	−0.03	−0.81 (0.418)
Unwanted Sexual Attention	0.13	0.01	0.41	7.13 (0.001)
*F*(df., df error)			36.07 *** (11, 338)	
*R*^2^			0.540	
Δ*R*^2^			0.526	
Δ*F*^2^			55.1 (0.001)	
Step 3				
Sex	0.003	0.007	0.01	0.37 (0.705)
Age	6.7	0.001	0.01	0.45 (0.648)
Professional category	−0.006	0.003	−0.08	−2.09 (0.037)
Scientific-academic field	0.000	0.002	−0.005	−0.14 (0.887)
Victim of sexual harassment	0.01	0.02	0.03	0.75 (0.453)
Victim of sex-based harassment	−0.01	0.01	−0.05	−1.06 (0.288)
Awareness of cases of sexual harassment	0.008	0.009	0.03	0.82 (0.410)
Awareness of cases of sex-based harassment	−0.005	0.01	−0.01	−0.42 (0.673)
Technology-facilitated sexual harassment behaviors	0.23	0.03	0.41	7.96 (0.001)
Gender Harassment	−0.006	0.008	−0.03	−0.74 (0.456)
Unwanted Sexual Attention	0.13	0.01	0.40	7.03 (0.001)
Knowledge of the protocol	0.001	0.007	0.004	0.10 (0.914)
Perceived effectiveness of the protocol	0.001	0.005	0.002	0.05 (0.954)
*F*(df., df error)			28.11 *** (14, 335)	
*R*^2^			0.540	
Δ*R*^2^			0.001	
Δ*F*^2^			0.055 (0.983)	

Note: *** *p* < 0.001.

## Data Availability

The data presented in this study are available from the corresponding author on reasonable request.
